# Military Hospital of Namur

**Published:** 1829-12

**Authors:** 


### MILITARY HOSPITAL OF NAMUR.

### *Case of Intermittent Inflammation of the Conjunctiva, cured by the Sulphate of Quinine.* By Dr. Fallot, of Namur.

A Swiss soldier, of robust constitution, was admitted the 21st of May, to be treated for inflammation of the eye. During his residence at Anvers, he had long suffered from the endemic intermittent fever of the place, and he had also contracted there a violent ophthalmia, which had destroyed the cilia of the left eyelid, and left a speck above the pupil.

On the morning after his admission, there was but little redness or pain of the eye. He was directed to bathe it with cold water. In the evening, the eye was violently inflamed, and very painful; the weakest light could not be endured. His pulse was quick, skin hot, tongue red, and great thirst. He was bled freely from the arm.

For several days the same intermission and recurrence of the symptoms had been observed. About four o'clock in the afternoon the eye began to be painful; the pains increased in severity for four or five hours, and then diminished, and gradually subsided; a copious perspiration at the same time covering the body.

M. Fallot preferred waiting for the occurrence of another paroxysm before he began the quinine, that he might be more certain he had formed a correct opinion from the account given him by the patient. The attack and subsidence of the symptoms took place just as before, and, after the termination of the paroxysm, two grains of the sulphate of quinine were given every hour. By this means the patient was cured. The eye was not again affected, and in a few days he was discharged.—*Journal Complémentaire.*

